# In-house development of an FPGA-based MCA8K for gamma-ray spectrometer

**DOI:** 10.1186/2193-1801-3-665

**Published:** 2014-11-10

**Authors:** Dang Lanh, Pham Ngoc Son, Nguyen An Son

**Affiliations:** Nuclear Research Institute, 01 Nguyen Tu Luc, Dalat, Vietnam; University of Dalat, 01 Phu Dong Thien Vuong, Dalat, Vietnam

**Keywords:** Field Programmable Gate Arrays (FPGA), Differential nonlinearity (DNL), Integral nonlinearity (INL)

## Abstract

The objective of this work is domestic development of electronics instruments. It used for measuring ionization radiation and practical training at Nuclear Research Institute (NRI), Dalat, Vietnam. The aim of this work is to study and develop a novel MCA8k for Gamma-ray spectrometer concerning experimental nuclear physics. An approach for design and construction of the aforementioned instrument is to apply logic integrating techniques via Field Programmable Gate Arrays (FPGA) under Max + PlusII, Altera. The instrument allows interfacing to PC with self-developed application software. Scientific significance of this work is partly to contribute to opening a research direction in the field of nuclear electronics science for design and construction of radiation measurement instruments with the advanced IC technology in Vietnam. Practical significance of this work is partly to contribute to enhancement of capabilities in developing radiation measurement instruments for experimental research as well as practical training in nuclear physics. The advantages of FPGA: overcoming ballistic deficit, decrement of serial and parallel noise, flexible in programming, control of the system by software without an interfere of hardware. The disadvantages of FPGA: requirement of good knowledge of VHDL and professional tools for development of a expected project. A new electronics module of MCA8k has been achieved. Some main results obtained from the experimental testing are as follows: differential nonlinearity (DNL) of FPGA-MCA8k approximately 1.27%, integral nonlinearity (INL) = 0.607%, time conversion ≈ 2.2 μs, deadtime (DT) is 0.75%. Data Acquisition Program MCANRI written in VC ^+ +^6.0, self-executed under Windows XP environment.

## 1. Introduction

So far, FPGA can be used in four main areas: digital signal processing, μC integration, interfacing among the entity classes and reconfiguration of design. Recently, the technological development of a new generation of electronics circuits and its role in application designs always show many highlights. The advantages of digital systems for gamma-ray spectrum in comparison with those of conventional electronics systems are reflected in the ability executing complex algorithms for signal processing (Redus 2009). According to this approach, the highest quality of measurements achieving at both low and high count rates as using different radiation detectors is possible. The main functions of a spectrometer as filtering and amplifying signals, detecting and eliminating overlapped pulses, analysis of amplitude and emission of energy spectrum (CAEN 2008), (Los Arcos and Garcia-Torano 1994) can be implemented effectively by digital algorithms using FPGA. The operations greatly increase the flexibility of the system, allow re-configuring and calibrating parameters without interfering the hardware (Bolic and Drndarevic 2002). In addition, the developed system can interface to PC easily. Recently, FPGA is used to develop nuclear instruments. These instruments are designed for high precision γ-ray spectroscopy with HPGe detectors and directly compatible with scintillator/PMT combinations: NaI, CsI, BGO, etc. As known, The DGF Pixie-4 is a multichannel data acquisition system for nuclear physics and other applications requiring coincident radiation detection (XIA LLC 2009), or the work for development of a fast flash ADC for nuclear spectroscopy system (Hien and Toshihiko 2001), or the issue for development of an FPGA based coincident system (Khang et al. 2013). Owing to a flexible inner structure in defining and changing the functions of electronics circuits by programming, FPGA expresses its capability for applying in gamma ray spectroscopy. Logic-logic linking method using IC FPGA in Max + Plus II environment with EPM7160E was spread in main procedures: forming a project and the initial conditions of design, handling project, generating information of graphic interface, compiling and loading data into a specific architecture. As results, FPGA contains the entire contents of the design and operates as a micro controller. In this work, the instrument is carried out by a logic element linking method. To develop nuclear electronics instruments under the direction of FPGA, a desired combination of hardware and software for performing algorithms of digital signal processing is current needs. Therefore, the following parts are presented: design and construction of MCA-8 k hardware, development of an application software for data acquisition and control of the instrument. On the other hand, the constructed instrument is able to interface to PC via a transferring-receiving port between peripheral devices and PC.

## 2. Design and method

### 2.1. Logic-logic combination method for integrated circuits of functional parts

**Figure 1:**
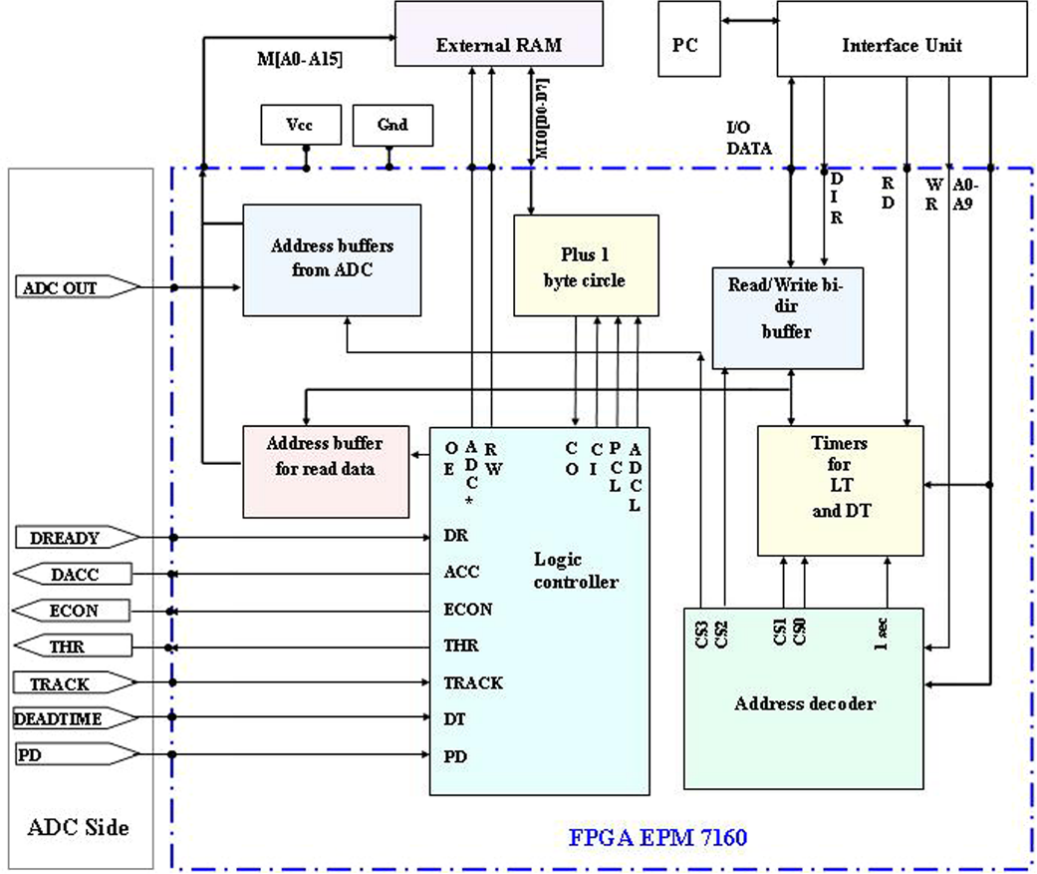
**A block diagram of FPGA-MCA8k configuration.**

**Figure 2:**
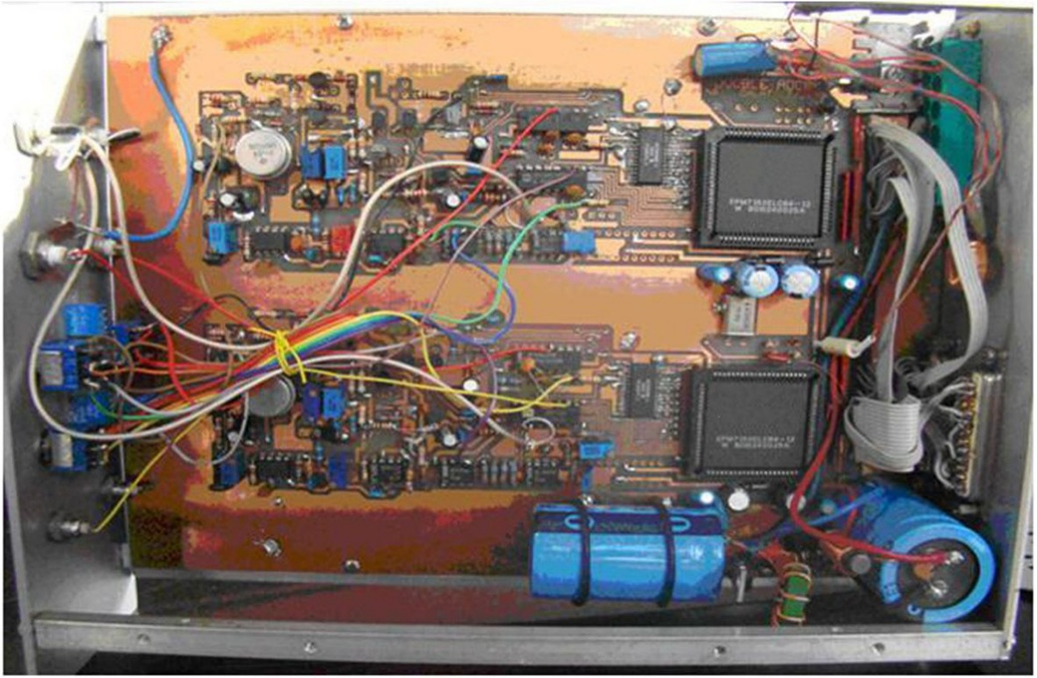
**Layout of FPGA-ADC8k.**

**Figure 3:**
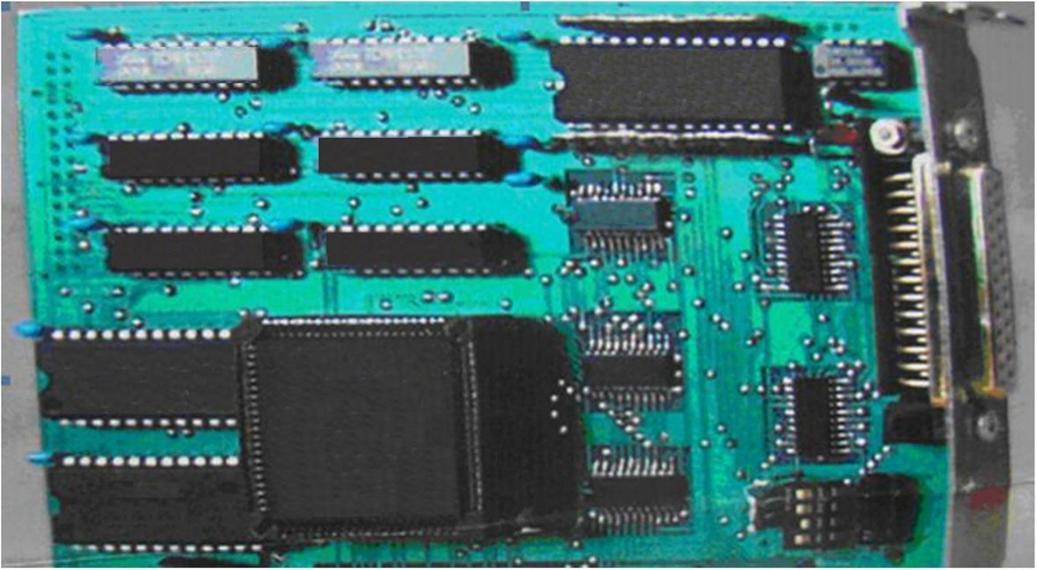
**Layout of FPGA-MCD8k.**

A block diagram of MCA (Multi Channel Analyzer) is presented in Figure 1. It consists of two main parts: ADC part and MCD (Multi Channel Data processing) part. Layout of FPGA-ADC8k in Figure 2 is ADC part. It includes the following main stages: input voltage follower (used LH0032G), pulse stretcher, peak detection and track/hold sample, analog to digital conversion (used AD7899BR-1), one FPGA EPM7160 for logic control as well as logic control. Layout of FPGA-MCD8k in Figure 3 is MCD part. It includes digital input buffers, digital output buffers, an external memory SRAM, 2 timers, and also one EPM7160 for logic control. Logic-logic combination method for integrated circuits of two parts is explained as follows. ADC part accepts a signal, converts it to a digital number, this number called a digital copy from the original analog signal. The copy will be carried out by MCD part. All of components for performance of electronics functions in MCD are formed inside an FPGA device, covered by a blue dash-line called the entity of the FPGA.

The instrument is constructed by FPGA application in which a logic processor is IC EPM7160E (a device of MAX 7000 family) with fast logic-logic combination about 5 ns (ALTERA 1998). The approach for setting data into FPGA entity was the professional method via parallel link. IC EPM7160E plays a role of internal logic controlling and data processing through all non-compact components. There are I/O ports allowing the FPGA to transfer external signals by each transaction either binary digits or TTL signals. In this study, the entity includes the following function elements: (i) A logic controller is responsible for the exchange of the question-response signals between MCD and ADC. (ii) Threshold conditions, peak detection, data output, and chip selection are carried out through a series of needed activities. As the asking-response condition between ADC side and MCD side following a clock sharing rule is satisfied, a conversion process starts and stores data into the external SRAM.

The main task of FPGA EPM7160E is integration of all digital parts as well as logic control parts for the both parts of ADC and MCD in the designed project carried out by integrated software environment (ISE) Max + PlusII. Since the particularity of the EPM7160E family is entirely digitized, all the analog components could not be embedded inside it. When the linear input follower is satisfying the threshold conditions for the question-response logic, a peak is detected and passed to the logic controller within the FPGA for handling, and SRAM contains spectral information. In principle, DPRAM can be created in an FPGA, but SRAM has to be used because the capacity of EPM7160E is not large enough. All other IC components are integrated in FPGA entity. Therefore, EPM7160E FPGA acts as the central data processor controlling the operation of the circuits by application software.

### 2.2. The processing unit and operation of the MCA8k

I/O ports of the entity allow the FPGA communicating with outside signals in every transaction. A logic controller is responsible for the exchange of the question-response signals between MCD and ADC. When DR (data ready) gives MCD a ready status to collect data, MCD returns two pulses (ACC (accepted) and ECON (enable conversion)) to ADC with variable thresholds establishing differentiated window for carrying out data acquisition. As the track signal (peak detection) appears, dead-time (DT) pulse allows the logic controller sending a pulse to the FPGA for timestamp (as a percentage). In parallel with the signal recognition logic and the question-and-response interface, the memory read/write function, living time (LT), dead time (DT) and real-time (RT) are done via other control logic pulses. To locate the address for SRAM write cycle, ADC pulse opens two byte buffers of 13-bit address. Peak amplitude content (reflecting energy information) existing in DR will be written on each cell of SRAM memory when OE (open enable) is low and RW (read-write) is high. Existing data on the local exchange line MIO [D0 - D7] written by each byte data via a series of internal logic control pulses ADCL (ADC latch), CI (Carry input), CO (Carry output) and ADC* (ADC ative low). To download the content contained in the SRAM, a memory read cycle is carried out by detecting low and high address with the decoded signal CS3. When two bytes of address read cycle have positioned SRAM, then contents will be read out via CS2. Data receiver-transmitters from peripheral to PC via bidirectional buffer the signal selected port (read or write) PSEL and choose the direction DIR signal effect. timers is programmable (2 bytes each) will be calculated from DT, LT clocks by 1 second. FPGA EPM7160E acts as the central data processor controlled the operation of the instrument by MCANRI.exe software applications.

### 2.3. The application software

**Figure 4:**
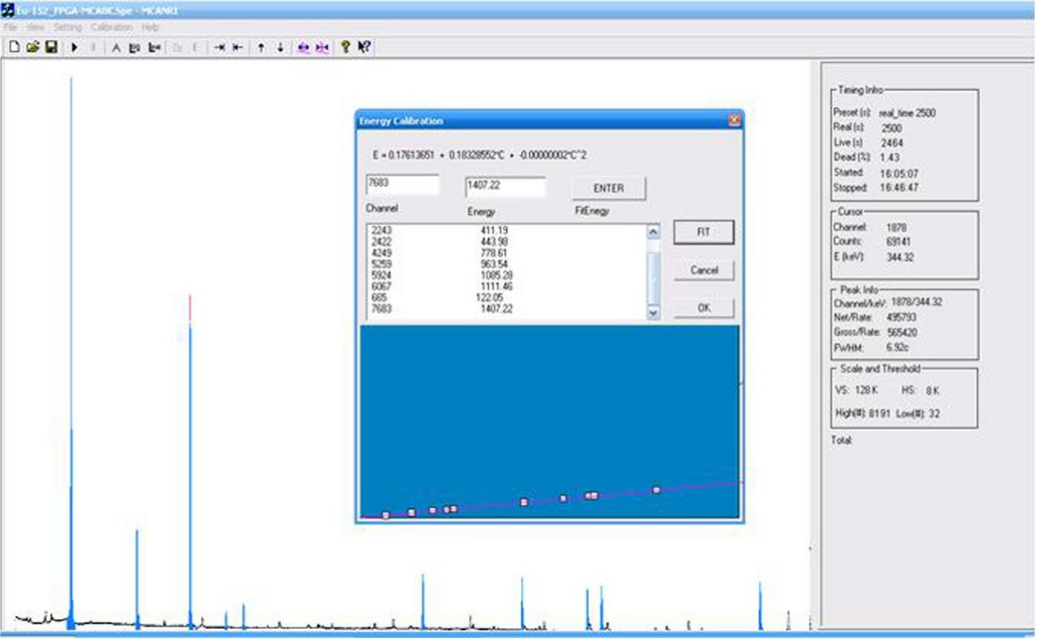
**The interface page of MCANRI program.**

**Figure 5:**
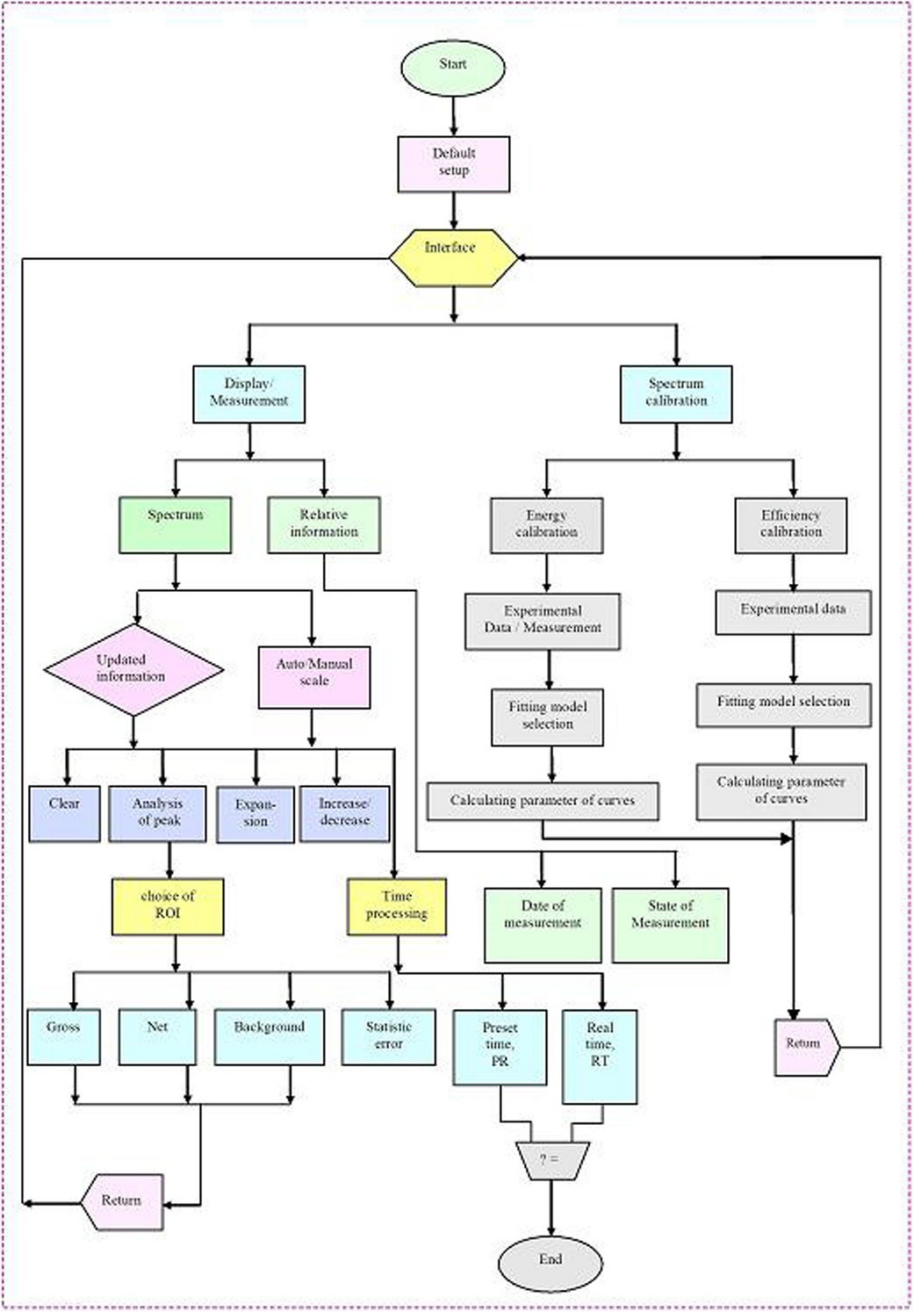
**The algorithm flowchart of MCANRI program.**

For controlling of data acquisition, setting of parameter and interfacing with user, an application program was developed, named MCANRI. The basic functions of the program are storing information of spectrum in PC memory devices, setting measuring parameters, and calibrations. The interface page and the algorithm flowchart are presented in Figures 4 and 5.

When the program begins, all default icons are displayed on the console screen. Two main branches of the flowchart are for measurement with peak analysis and energy/efficiency calibrations. As satisfying a combination of the instrument, spectrum and timing information are displayed. The spectrum is displayed and updated every millisecond in auto scale or manual mode.

**Figure 6:**
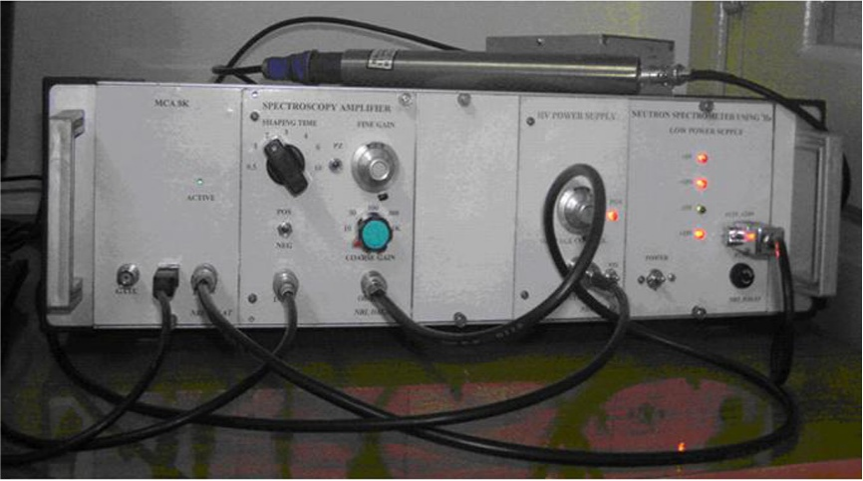
**The neutron counting system used at the horizontal channel of the reactor, NRI, Dalat.**

Besides, the application program MCANRI is not only used for data acquisition in gamma spectrometer, but also used for the neutron counting system using ^3^He detector at horizontal experiment channels of the reactor, Nuclear Research Institute (NRI), Dalat. Figure 6 shows the neutron counting system.

## 3. Experimental measurements and results

### 3.1. Determination of differential nonlinearity of MCA

**Figure 7:**
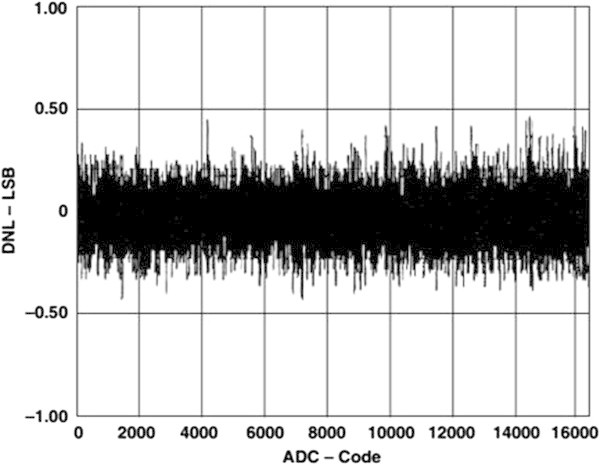
**Typical DNL plot for the AD7899.**

**Figure 8:**
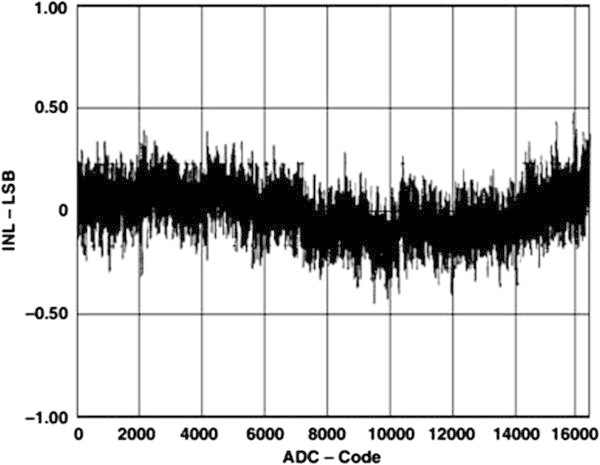
**Typical INL plot for the AD7899.**

One of the largest contributions to the DNL/INL of the FPGA-MCA system is coming from the ADC. As constructing the system, AD7899 from ANALOG DEVICES (Analog Devices, Inc. 2001) is used. The AD7899 is a fast, low-power, 14-bit A/D converter that operates from a single 5 V supply. The part contains a 2.2 μs successive-approximation ADC, a track/hold amplifier, 2.5 V reference, on-chip clock oscillator, signal conditioning circuitry, and a high-speed parallel interface. The part accepts analog input ranges of ±10 V. The plots in Figures 7 and 8 show typical DNL and INL for the AD7899, respectively.

**Figure 9:**
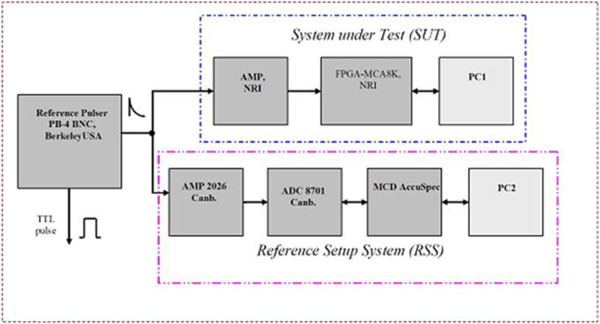
**Configuration of measurement for differential nonlinearity of MCA.**

**Figure 10:**
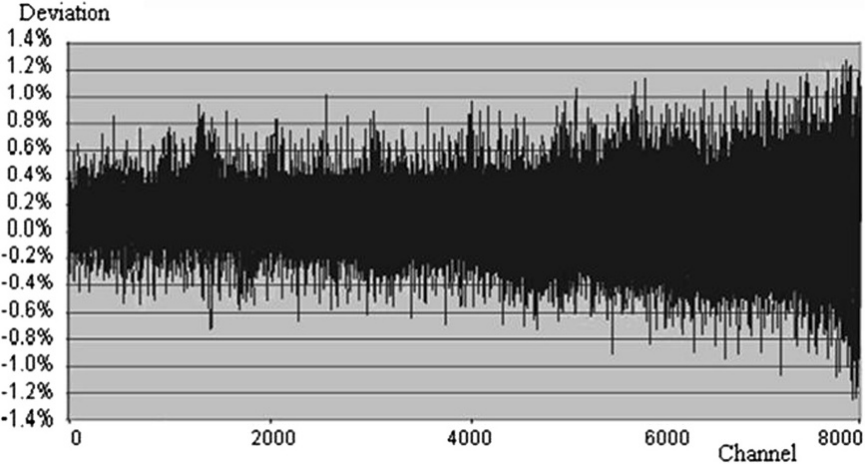
**Differential nonlinearity of MCA in SUT.**

**Figure 11:**
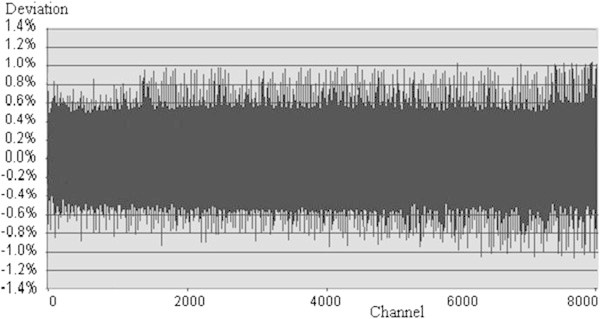
**Differential nonlinearity of MCA in RSS.**

To test differential nonlinearity of MCA (DNL_FPGA-MCA_), an experimental arrangement is performed as shown in Figure 9. This configuration consists of two independent measurement branches: the upper branch of the system (system under test - SUT) is to test the technical characteristics of the instruments formed from AMP-NRI (Hien and Lanh 1988), FPGA-MCA8k, computer 1, data collection program MCANRI; the below branch (reference setup system - RSS) includes AMP 2026 Canberra, ADC 8701 Canberra, MCD Accuspec V1.1, software MCA Series 100 and computer 2. Saw-generator type LG-1 BNC, Berkeley, USA controls signal amplitude in random pulser DB-2 BNC, Berkeley, USA. Shaping time of both AMPs is chosen 4 μs to reduce the effect of rising time as using the reference pulser. The experiment gives counts for full range of 8192 channels:  leading to the average number of counts: N_av_ ≈ 179979.143. From N_av_ to find the maximum deviation value in 8192 channels: ΔN_max_ = (N_x_ - N_av_)_max_ = 2285.735. Thus, differential nonlinearity (IAEA 2008) of the FPGA-MCA8k is: DNL_FPGA-MCA8K_ = (2285.735/179979.143) × 100% ≈ 1.27%. Statistical fluctuation of counts in Figure 10 represents its differential nonlinearity for SUT. Similar to the above-mentioned calculation, differential nonlinearity obtained in RSS (please refer to Figure 11): DNL_RSS_ = 1.03%. Also, deadtime in both systems, respectively: DT_FPGA-MCA8K_ = 0.56% and DT_RSS_ = 0.47%. All results are shown in Table 1.

**Table 1:** **Experimental results of the integral nonlinearities of MCAs**

Instruments	T_meas._(s)	V_in_(mV)	Mode	τ_AMP_(μs)	Range (#)	Counts	DT (%)	DNL (%)
RSS_Accuspec_	36000	10^4^	PHA	4	8192	179154	0.47	1.03
SUT_FPGA-8K_	36000	10^4^	PHA	4	8192	178972	0.56	1.27

In principle, both of results from RSS and SUT must be the same. However, this result is different from the other. SUT’s result is worse than that of RSS. Please refer to Figures 10 and 11. It is clear that the quality of designed circuits of SUT is not better than that of RSS. Because statistical fluctuation of counts in higher channels is bigger than statistical fluctuation of counts in lower channels, therefore deviations of counts in higher channels must be larger. This reason makes spectrum in higher channels bigger, it means that there is an increment for DNL.

When the specific technical parameters of the MCA are tested, a reference setup system (RSS) as a basis for evaluating operating modes as well as the reliability of the execution method demonstrated through experimental data measurements. Table 1 showed that the deviation between differential nonlinearity of FPGA-MCA8k and that of MCD Accuspec is  In comparison with differential nonlinearity of MCD-Accuspec, differential nonlinearity of FPGA-MCA8k is larger than that of MCD-Accuspec (1.27 + 0.233) – 1.03 = 0.473. Although differential nonlinearity of FPGA-MCA8k does not achieve the nominal standard, it is still acceptable for radiation measurements and training purpose. In the reality, it affects the channel widths in analog to digital conversion, and variation of widths leads a high fluctuation of counts.

### 3.2. Determination of integral nonlinearity of MCA

**Figure 12:**
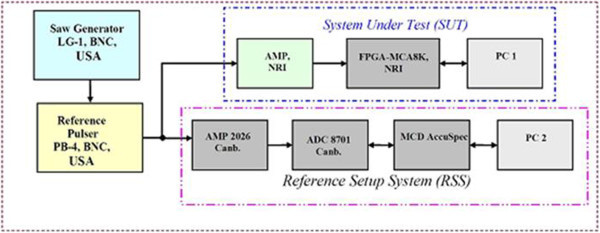
**Configuration of measurement for integral nonlinearity of MCA.**

To test integral nonlinearity of MCA (INL_FPGA-MCA_), an experimental configuration is performed in Figure 12. Two measurement programs are MCANRI and MCA Series 100. Reference Pulser PB-4 BNC, Berkeley, USA generates a positive nuclear tail pulse with rise time of 25 ns and falling time of 50 μs. Set thresholds of LLD ≈ 16 mV, ULD maximum of 10000 mV. Changing the incremental steps from 0 to 10000 mV, test steps are 40. The corresponding channel-voltage value pairs listed in the Table 2. From the experimental fitting function of y = 0.82254× – 12.3832, where x is the amplitude of the input signal, y is the estimated channel, −12.3830 is the amplitude at channel zero, 0.82250 is the slope of the fit line and coefficient of determination R^2^ = 0.9997 (please refer to Figure 7), replacing X_i_ = (17 ÷ 9872) with i є [1, 40], then 40 values of Y_i_ is obtained. Therefore, *ΔY*_max_ *=* (Y_*r*_*– Y*_*i*_)_max_ = 49.36 and *Y*_*max*_ = 8132. According to reference ( ORTEC, CAMAC ADCs and Memories), integral nonlinearities in SUT and RSS are, respectively, calculated as follows:

**Table 2:** **Results of integral nonlinearity of the MCA**

No.	Voltage X_i_(mV)	Y_r_	Y_i_	ΔY_max_	No.	Voltage (mV)	Y_r_	Y_i_	ΔY_max_
1	17	18	1.59998	16.40002	21	4962	4089	4068.329	20.67068
2	191	158	144.7219	13.27806	22	5219	4259	4279.624	−20.62444
3	431	327	342.1315	−15.13154	23	5469	4501	4485.164	15.83556
4	690	568	556.0618	11.9382	24	5707	4659	4680.839	−21.83852
5	930	745	753.3802	−8.3802	25	6032	4956	4948.041	7.95948
6	1188	971	965.4975	5.50252	26	6270	5149	5143.715	5.2854
7	1440	1199	1172.682	26.3182	27	6484	5293	5319.657	−26.65684
8	1699	1388	1385.621	2.37876	28	6749	5576	5537.529	38.47076
9	1938	1584	1582.117	1.88252	29	7002	5767	5745.536	21.46428
10	2190	1817	1789.302	27.6982	30	7342	6042	6025.07	16.92988
11	2450	2001	2003.063	−2.0634	31	7584	6127	6224.033	−97.03284
12	2696	2219	2205.315	13.68524	32	7973	6574	6543.853	30.14692
13	2950	2408	2414.143	−6.1434	33	8242	6727	6765.014	−38.01412
14	3202	2456	2621.328	−165.3277	34	8522	7034	6995.219	38.78108
15	3452	2851	2826.868	24.13228	35	8700	7102	7141.563	−39.5634
16	3703	3023	3033.23	−10.22988	36	9162	7532	7521.401	10.59868
17	3965	3298	3248.636	49.3642	37	9320	7659	7651.303	7.6974
18	4216	3467	3454.998	12.00204	38	9497	7798	7796.825	1.17508
19	4485	3661	3676.159	−15.159	39	9837	8114	8076.359	37.64068
20	4716	3879	3866.078	12.92204	40	9872	8132	8105.135	26.86508



**Figure 13:**
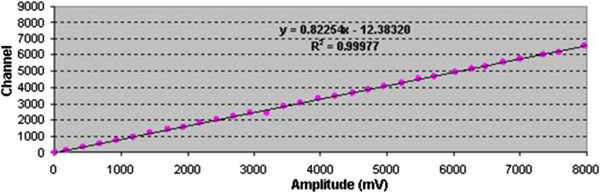
**Integral nonlinearity of the MCA.**

The Integral nonlinearity INL_FPGA-MCA8K_ is about 4 times bigger than INL_MCD8K-Accuspec_; therefore, integral nonlinearity of the instrument is worse as higher energy. On the other hand, as presented in Figure 13, the signal amplitude is strong correlation with the corresponding channel within statistical fluctuation. It could be estimated that electronics noise and/or unstable baseline restorer are the basic reasons of the difference between the MCA8k and the standard one. In other word, DNL/INL affect the channel width distributions and uncertainties of counts in the system. As results, the higher the energy, the worse the resolution.

### 3.3. Test of count accuracy and data throughput of the MCA

Accuracy of count numbers of FPGA-MCA8k was determined by an experimental configuration in Figure 9 using TTL pulse output. Frequency generator is changed in a range from f_min_ = 90 Hz to f_max_ = 300 kHz, for both of RSS and SUT systems, measuring in PHA mode with preset time t_pr_ = 10000 s. The deviation of RSS and SUT was calculated (CANBERRA 2009) by the formula:  where C_r_ is counts obtained in RSS and C_t_ is counts in SUT. When elapsed time is equal to preset time t_pr_, both systems automatically stop. Accumulation of counts against time and their differences between two systems are shown in Table 3.

**Table 3:** **Accumulated counts versus time and their differences between two systems**

Test times	Time of measurement	Frequency	Counts, C_r_, in RSS	Counts, C_t_, in SUT	Deviation D% (%)
1	t_pr_ = 10000 s	f_min_ = 90 Hz	899075	898526	0.0611
2	t_pr_ = 10000 s	f = 500 Hz	4975124	4973392	0.0348
3	t_pr_ = 10000 s	f = 1 kHz	9938031	9930125	0.0796

In Table 3, test results in terms of counting accuracy and frequency of input–output data of the present designed MCA show the average deviation of 0.06 in the frequency range of 90 Hz – 1 kHz, in comparison with the standard case.

### 3.4. Test of spectrum calibration and peak analysis

**Figure 14:**
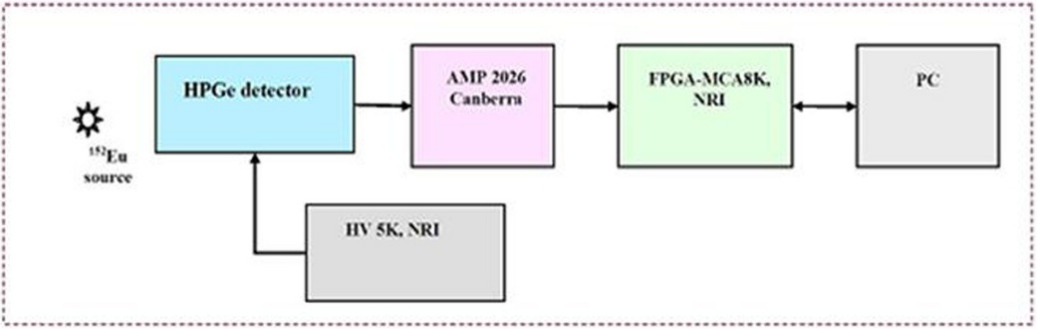
**An experimental spectrometer for measuring gamma spectrum from** ^**152**^ **Eu source.**

A spectrometer for measuring gamma ray emitted from ^152^Eu source was setup in Figure 14. C2019 type HPGe detector, Inter-techniques is fed to all functional electronics modulars: AMP 2026-Canberra, FPGA-MCA8k, HV 5 k- NRI, and PC. Application software for getting data is MCANRI. All parameters setup with MCANRI and timing information are as follows:

HPGe C2019 detector, Intertechniques: positive HVPS = 2500 V, resolution 2.05 keV at peak 1332.5 keV of ^60^Co, t_real_ = 2500 s, LL = 32, UL = 8191.

When t_live_ = t_preset_ = 2500 seconds, MCANRI program automatically stops; ^152^Eu spectrum displays on screen in Figure 4. It allows user performing all following procedures: Energy calibration using quadratic curve, peak area calculation P, gross G, background B, and standard deviation σ_p_. Refer to Figure 4, all following information are derived: preset time is equal to real time being 2500 s, elapsed time is 2464 s, dead time is 1.43%; starting at 16:05:07 and stopping at 16:46:47; the main pointer at central energy 344.32 keV with 69141 counts according to channel 1878. Lower level is 32, upper level is 8191, vertical scale is 128 k, range of measurement is 8 k. As entering 10 experimental ‘channel-energy’ values and fitting, the experimental quadratic energy calibration curve (keV) is a function of channel (*C*):


After calibrating energy, making ROI (region of interest) of peak is the next step; at that time, peak area, background, gross, standard deviation and energy resolution are calculated (IAEA 1991), respectively. ^152^Eu spectrum and the energy calibration curve via a fit function of ten peaks with energy-channel value pairs is shown in Figure 4. Table 4 shows the experimental results.

**Table 4:** **The quantitative values of ten-experiment gamma-ray energy peaks from** ^**152**^ **Eu**

Gama-ray energy from Eu-152 E (keV)	Emission probility P_γ_(%)	MCANRI energy calibrated E (keV)	FWHM (keV) for SUT	FWHM (keV) for RSS	Net area of peak	Measured efficiency (%)	Calibrated fitting efficiency (%)	Deviation (Measured-Fitting)
121.78	28.58	122.05	1.11	1.07	908937	1.03	1.029	0.001
244.69	7.58	245.01	1.24	1.19	176315	0.75	0.768	−0.018
344.28	26.50	344.50	1.27	1.25	495793	0.67	0.619	0.051
411.11	2.234	411.19	1.54	1.50	35394	0.51	0.544	−0.034
443.96	3.148	443.98	1.65	1.59	49876	0.51	0.513	−0.003
778.90	12.942	778.79	1.68	1.63	139620	0.35	0.349	0.001
964.07	14.605	963.54	1.84	1.81	144575	0.32	0.316	0.004
1085.87	10.207	1085.28	1.91	1.86	100528	0.32	0.298	0.022
1112.07	13.644	1111.48	1.99	1.92	114867	0.27	0.294	−0.024
1408.00	21.005	1407.22	2.10	2.07	156054	0.24	0.239	0.001

## 4. Conclusions

This work carried out a design and construction of an FPGA-based MCA8k. FPGA EPM7160E acts as the central data processor controlled operations of the instrument by MCANRI software application. The integrated software environment for development of the entity FPGA is Max + PlusII, Altera. Main technical characteristics are as follows:

- FPGA-MCA8k interfacing to PC via the parallel port (LPT),
- Resolution: 8192 channels,
- Time conversion: approximate to 2.2 μs,
- The Integral nonlinear INL_FPGA-MCA8K_ ≈ 0.607% of the full scale,
- The Differential nonlinear DNL_FPGA-MCA8K_ ≈ 1.27% over the measurement range,
- Maximum count capacity per channel is 16777215,
- The low and up levels for the ADC are controlled by software,
- Input receiving positive, unipolar pulse peak amplitude from 0 to 10 V,
- Data Acquisition Program MCANRI written in VC ^++^6.0, self-executing under Windows XP environment.

Field Programmable Gate Arrays (FPGA) technique has been successfully applied for development of a new MCA unit by means of logic-logic combination using the integrated software environment Max + plusII of Altera. This study creates a potential of low cost electronics module for gamma-ray spectrometers at Nuclear Research Institute, Dalat, Vietnam. In this study, we have designed, fabricated, tested and adjusted the new MCA module for technical parameters to meet requirements of practical applications. There are I/O ports allowing the FPGA entity to transfer external signals by each transaction either binary digits or TTL signals. It includes the following function elements: (i) A logic controller is responsible for the exchange of the question-response signals between MCD and ADC. (ii) Threshold conditions, peak detection, data output, port selection, and chip selection are carried out through a series of needed activities. This MCA module is properly interfacing to PC through LPT or USB port. Regarding software, an application program called MCANRI has been developed by the VC^++^6 compiler. This version of program contains almost basic functions required in radiation spectrum measurements, such as energy, efficiency calibrations, peak analysis, and timing parameter setup. In addition, it can be applied for data processing to test the technical characteristic parameters of the fabricated instrument and calculate the sufficient physics quantities in spectroscopy experiments measuring ionization radiation.

## Abbreviations

ADC: Analog to digital converter
DC: Direct current
DIR: Direction
FWHM: Full-width half-maximum
I/O: Input/Output
LLD: Low level discrimination
MIO: Memory input–output
PCL: Port controlling link
PSEL: Port selection
RSS: Reference setup system
RW: Read-write
SRAM: Static random access memory
SUT: System under test
ULD: Upper level discrimination.
